# Decline of long-range temporal correlations in the human brain during sustained wakefulness

**DOI:** 10.1038/s41598-017-12140-w

**Published:** 2017-09-19

**Authors:** Christian Meisel, Kimberlyn Bailey, Peter Achermann, Dietmar Plenz

**Affiliations:** 1 Section on Critical Brain Dynamics, National Institute of Mental Health, Bethesda, Maryland 20892 USA; 2 Department of Neurology, University Clinic Carl Gustav Carus, Fetscherstraße 74, 01307 Dresden, Germany; 3 Institute of Pharmacology and Toxicology, University of Zurich, Winterthurerstrasse 190, 8057 Zurich, Switzerland

## Abstract

Sleep is crucial for daytime functioning, cognitive performance and general well-being. These aspects of daily life are known to be impaired after extended wake, yet, the underlying neuronal correlates have been difficult to identify. Accumulating evidence suggests that normal functioning of the brain is characterized by long-range temporal correlations (LRTCs) in cortex, which are supportive for decision-making and working memory tasks. Here we assess LRTCs in resting state human EEG data during a 40-hour sleep deprivation experiment by evaluating the decay in autocorrelation and the scaling exponent of the detrended fluctuation analysis from EEG amplitude fluctuations. We find with both measures that LRTCs decline as sleep deprivation progresses. This decline becomes evident when taking changes in signal power into appropriate consideration. In contrast, the presence of strong signal power increases in some frequency bands over the course of sleep deprivation may falsely indicate LRTC changes that do not reflect the underlying long-range temporal correlation structure. Our results demonstrate the importance of sleep to maintain LRTCs in the human brain. In complex networks, LRTCs naturally emerge in the vicinity of a critical state. The observation of declining LRTCs during wake thus provides additional support for our hypothesis that sleep reorganizes cortical networks towards critical dynamics for optimal functioning.

## Introduction

Sleep is essential for daytime functioning and well-being. Without sleep optimal brain functioning such as responsiveness to stimuli, information processing, or learning is impaired^1–4^. The neuronal correlates and mechanisms by which sleep improves, or, conversely, by which the lack of sleep impairs cognitive function and information processing in cortical networks, are largely still not understood.

An essential ingredient for information processing is thought to be the ability of neural circuits to integrate information over extended periods of time. For example, in decision-making and working memory tasks^5–8^, this ability may increase the signal-to-noise ratio and afford to maintain some memory of past activity. The network dynamics typically observed with this ability are characterized by slowly decaying autocorrelation functions, or, in general, long-range temporal correlations (LRTCs). Accordingly, slow autocorrelation decays have been observed using different experimental modalities, including studies on non-human primates^9,10^, human electrocorticography^11^ as well as fMRI^12^. In a large body of EEG research, slow timescales associated with long-range temporal correlations have been observed and studied in the amplitude fluctuations of ongoing neuronal oscillations^13–21^. Together, these studies provide strong support for the existence of slow timescales associated with LRTCs in cortical activity and their important role for the integration of information in brain networks.

The link between LRTCs and information processing is further supported by the insight that LRTCs are generic features of a critical state^22^. A critical state is a state at a higher-(second-) order phase transition where the long-term qualitative behavior of a system changes rapidly from one dynamical phase to another. In networks, for example, a critical state can be observed at the onset of persistent network activity or at the transition from asynchronous to synchronous activity. Being at or close to such a critical state (i.e. close to criticality) has been shown to provide advantageous features for network computation and information processing^23–26^. Numerous computational and experimental studies^24,26–31^ have provided support for the hypothesis that also brain networks operate at or near a critical state and thereby take advantage of the computational capabilities provided by criticality. In this context, the observation of LRTCs in EEG dynamics has been taken as supporting evidence for the criticality hypothesis^13^.

In a previous study, we found various signatures of critical dynamics to fade over the course of sleep deprivation^32^. These observations led to the hypothesis^32^ that deviations or disruptions of critical dynamics and its advantageous features for network computation might underlie the impaired cognitive functioning observed during sleep deprivation^2^. The question whether LRTCs are similarly affected in a wake-time dependent manner, however, is still open.

Here, we systematically characterize the LRTCs governing cortical dynamics during extended wake. We find that the initially slow timescales characterizing the autocorrelation function hours of sustained wakefulness. Details are progressively shortened as sleep deprivation progresses. This decline becomes evident when taking changes in EEG signal power into appropriate consideration. The results support a hypothesis on the network function of sleep, to re-organize cortical networks towards critical dynamics with long-range temporal correlations for optimal function during wake.

## Materials and Methods

### EEG recordings during prolonged wakefulness

We analyzed wake electroencephalogram (EEG) recordings of eight healthy young right-handed males (23.0 ± 0.46 years; mean ± s.e.m.) over the course of 40 hours of sustained wakefulness. Details on the conduction of the experiment are provided in the original publications^33,34^. Participants spent the sleep deprivation period in the sleep laboratory and its surroundings and were under constant surveillance by a member of the experimental team. Lab temperature was approx. 20 °C, with normal indoor light. Participants were engaged in studying, playing games, watching films and occasionally taking a walk outside the laboratory. Meals were scheduled at 07:30, 12:00 and 18:00, and they took a shower at 08:00. Caloric content was not controlled. Three days prior and during the entire experiment participants had to abstain from caffeine consumption. All participants were low to moderate coffee consumers. At the time when the wake EEG recordings were performed, it was the sixth day without caffeine consumption. As they were low to moderate consumers we do not expect withdrawal symptoms at this point in time. Participants were recruited among university students. They underwent a screening night with polysomnograpic recordings in the sleep laboratory. Exclusion criteria were the presence of sleep disturbances such as sleep apnea and nocturnal myoclonus, prolonged sleep latency and low sleep efficiency. Use of any medication was an exclusion criterion. For the three days prior to the experimental session, as well as during the whole experiment, the subjects were instructed to maintain a regular sleep-wake cycle with sleep scheduled from 23.00 to 07.00. Compliance with the latter instruction was verified by ambulatory activity monitoring.

Waking EEG over the course of sleep deprivation was recorded every three hours over 14 sessions, starting at 07:00. Another waking EEG was recorded after a recovery night of sleep, totaling 15 EEG sessions in all. Sessions consisted of a 5 min eyes-open period, followed by a 4–5 min eyes-closed period and a final 5 min eyes-open period. Twenty-seven EEG derivations (extended 10–20 system; reference electrode 5% rostral to Cz) were sampled at 256 Hz (high-pass filter at 0.16 Hz; anti-aliasing low-pass filter at 70 Hz). During the eyes open condition, they had to fixate on a black dot at the wall. For both conditions, they were instructed to try to avoid eye blinks. Light was on during the wake EEG recordings. Participants were continuously monitored during EEG recordings. When signs of drowsiness were detected (e.g. closure of the eyelids, slow, pendular eye movements and eye blinks), the subject was addressed by the experimenter and asked to respond. Apart from EEG, only subjective alertness was additionally assessed, which is reported in refs ^32,33^.

### Preprocessing of EEG signals

Artifacts including eye blinks were marked by visual inspection. All analyses were performed on artifact-free signal segments during the eyes open condition. Electrodes were re-referenced to average reference. Segments for further analysis were chosen to be 20 seconds long (5120 samples) as a balance between, on one hand, including many segments in the analysis and, on the other hand, having long enough, continuous segments to assess timescales related to long-range temporal correlations (LRTCs). An average of 18 20-s segments corresponding to a total of 6 min of EEG data from both eyes open conditions was analyzed in each subject and EEG session.

### Signal power

**Figure 1:**
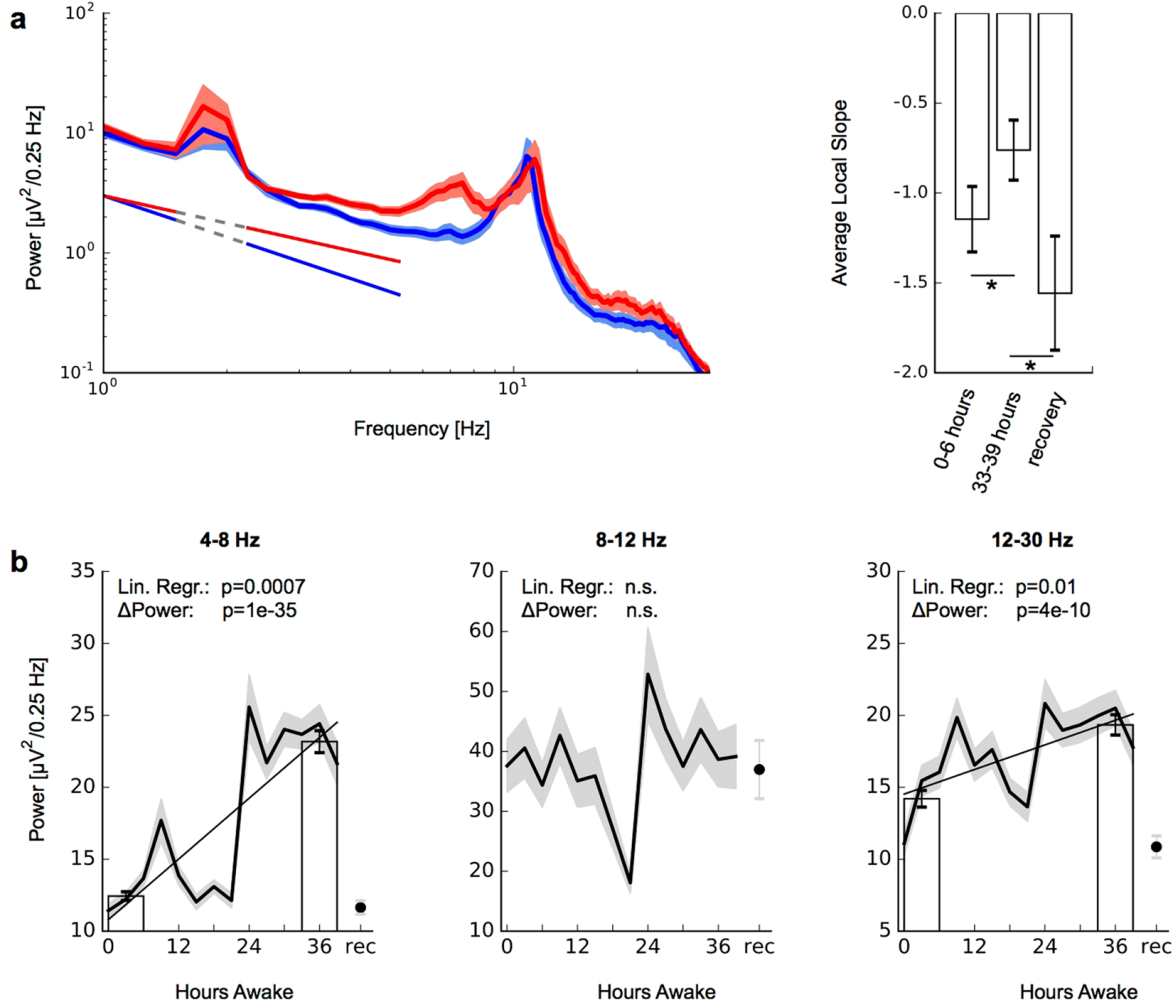
Changes in EEG power during sleep deprivation. (**a**) EEG power density at the beginning of sleep deprivation (0–6 hours, blue) and end of sleep deprivation (33–39 hours, red; grand average across 8 subjects and all channels, error bars indicate s.e.m.). Local slopes of the PSD were obtained in the ranges indicated by the solid lines. Right: Local slopes indicate a more shallow PSD at the end of sleep deprivation compared to beginning of sleep deprivation and after recovery sleep. (**b**) Power changes in frequency bands. Straight black lines indicate linear regression results; bars indicate the average power values at the beginning (0–6 hours) and at the end (33–39 hours) of sleep deprivation. Of the three frequency bands, only the alpha band (8–12 Hz) showed no significant change during sleep deprivation as judged by linear regression and difference between bars (ΔPower).

Power analyses were done on the same artifact-free 20-s segments used in the timescales analyses to assess LRTCs. For each frequency band of interest (4–8 Hz, 8–12 Hz, 12–30 Hz), EEG power density spectra were computed (FFT routine; 4 s, non-overlapping Hanning window). The power value for each 20-s interval and channel was then obtained as the sum across the frequency band of interest. Local slopes of the power spectral density (Fig. 1) were computed across lower frequencies in double-logarithmic coordinates.

### Estimation of long-range temporal correlations in EEG

We here investigate the structure of EEG temporal correlations by means of autocorrelation and detrended fluctuation analysis (DFA). There exists a direct relationship between autocorrelation, the scaling exponent derived from detrended fluctuation analysis and the power spectrum of a signal^35–37^. Intuitively, the autocorrelation function measures how similar the signal is with itself at a later time. A signal is said to exhibit long-range temporal correlations, if the autocorrelation decays according to a power-law (with an exponent between −1 and 0) as a function of time. This slow decay guarantees a positive autocorrelation (or more intuitively: some memory about the past) even for large time lags. In practice, however, the estimation of the autocorrelation functional form and its exponent may be complicated since the autocorrelation function can be affected by trends and often becomes very noisy in its tail for large time lags. Detrended flucuation analysis overcomes these problems and allows a more robust estimation of LRTCs^36^. Nevertheless, the intimate relationship between autocorrelation and DFA posits that both must change accordingly when the underlying temporal correlation structure of a signal changes. We therefore always analyze both the autocorrelation and DFA in EEG throughout this study to conclusively assess changes in LRTCs over the course of sleep deprivation.

#### Autocorrelation

Autocorrelation functions were derived from the envelope of ongoing oscillations in EEG signals in confined frequency bands. Specifically, artifact-free EEG signals of 20-s duration were filtered in the respective frequency band (theta: 4–8 Hz; alpha: 8–12 Hz; beta/low gamma: 12–30 Hz; phase neutral filter by applying a third order Butterworth filter in both directions) and the signal envelope was derived using the absolute value of the Hilbert transform. The autocorrelation function *ACF*(*s*) of the envelope signal *x*(*t*) with length *N*, mean *μ* and variance *v* was then derived by

1 $$ACF(s)=\frac{{\sum }_{t=1}^{N-s}(x(t)-\mu )\,(x(t+s)-\mu )}{v},\quad s=1,\ldots ,N/2.$$

The speed by which the autocorrelation function decays thereby provides information about the persistence of temporal correlations in the signal. We here quantified the autocorrelation function decay by capturing its value at lag one, i.e. *s* = 1 in equation 1. Lag-1 autocorrelation is a frequently used measure to robustly estimate the autocorrelation function decay in many dynamical systems (for review see refs ^38,39^ and paper cites therein) and has also been applied to neurophysiological data^40^. As an alternative, we also quantified the autocorrelation function decay by capturing the first lag-value where the autocorrelation function was equal or below 0.5. This method too has previously been used in neuroscience research to quantify the autocorrelation function decay^11^. Both methods of quantification revealed similar results.

#### Detrended fluctuation analysis

As a second approach to assess the autocorrelation structure in our data, we applied detrended fluctuation analysis (DFA). DFA has been applied numerous times in EEG to quantify LRTCs before, see ref. ^36^ for a review. For each artifact-free, filtered (theta: 4–8 Hz; alpha: 8–12 Hz; beta: 12–30 Hz; phase neutral filter by applying a third order Butterworth filter in both directions), 20-second signal segment, we first extracted the absolute value of the signal’s Hilbert transform. This provided the amplitude envelope of the signal *x*. Next, we determined the signal profile *Y*(*i*) by subtracting the mean of the signal and computing its cumulative sum:

2 $$Y(i)=\sum _{k=1}^{i}\,{x}_{k}-\langle x\rangle $$

All subsequent steps were performed on the profile of the signal.

We defined a set *T* of window sizes on a logarithmic scale. These window sizes were equally spaced between 16 sampling points (0.0625 s) and 4096 sampling points (16 s). This range is a compromise between, on one hand, computing the average fluctuation function for a given window size from many signal sub-segments on the one side, and, on the other hand, having adequate samples in each subsegment over which to fit a least squares regression in the detrending step on the other side^36^.

Next, the fluctuation function *F*(*t*) for each window size *t*∈*T* was found using the following three steps. First, the profile was split into *N* non-overlapping sub-segments of length *t*. Second, we fit a polynomial of a given, fixed order to each sub-segment and subsequently subtracted the fit from that sub-segment. The detrended sub-segment *Y*_*d*_(*t*) of length *t* is thus:

3 $${Y}_{d}(t)=Y(t)-{p}_{v}(t)$$

where *p*_*v*_(*t*) is the polynomial fit of the *v* th sub-segment. Third, the standard deviation was calculated for each detrended sub-segment. *F*(*t*) for a given window size *t* was then calculated as the average standard deviation across all sub-segments of size *t*.

Finally, *F*(*t*) was plotted for all window sizes on log-log axes. If a time series is characterized by long-range temporal correlations, then *F*(*t*) increases with window size *t* according to the power law:

4 $$F(t)\propto {t}^{\alpha }$$

Thus, from this log-log plot, the DFA scaling exponent (or Hurst exponent) can be estimated as the slope *α* via linear regression. *α* > 0.5 indicates long-range correlations, *α* < 0.5 indicates an anti-correlated signal and *α* = 0.5 indicates an uncorrelated signal.

**Figure 2:**
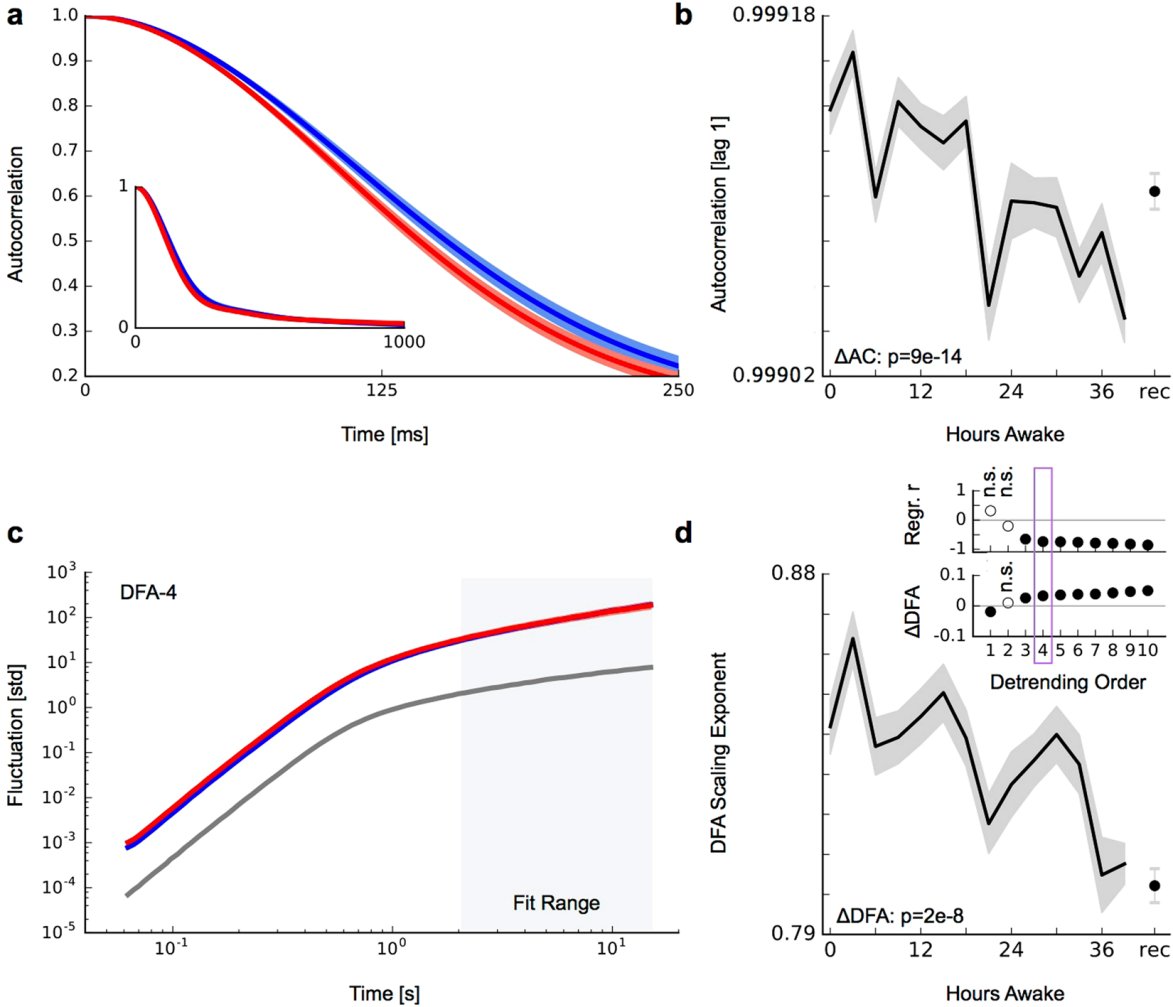
Decline in signal autocorrelation and long-range temporal correlations during sleep deprivation. (**a**) Autocorrelation function of the signal envelope in the alpha band (0–6 hours, blue; 33–39 hours, red; inset, autocorrelation functions until 1 s). (**b**) Faster autocorrelation decay during sleep deprivation (0–39 hours of sleep deprivation and after consecutive recovery sleep, rec). The solid black line corresponds to the mean across all channels from all 8 subjects, error bars indicate s.e.m. (**c**) Detrended fluctuation analysis of the of the signal envelope in the alpha band. Surrogate white-noise data are depicted in grey. DFA scaling exponents were obtained from linear fits in double-logarithmic coordinates aver the depicted fitting range. (**d**) Decline in long-range temporal correlations estimated by the DFA scaling exponent. The inset shows results for different polynom detrending orders. Results settle for detrending order of three and higher as judged by linear regression analysis and the difference between early (0–6 hours) and late (33–39 hours) sleep deprivation (ΔDFA).

To accurately estimate the DFA scaling exponent, it is necessary to choose the proper range of window sizes over which to perform linear regression. Filtering can introduce short-range correlations and cause an overestimation of the exponent, if included^36^. To determine the effect of our filters, we simulated a set of 100 white noise segments of the same length as our analyzed signal segments (20 s at 256 Hz sampling), applied each of our bandpass filters to the set, computed the average fluctuation function for each window size and plotted the log-log DFA plot. This kink visible in this plot demonstrates the impact of filters on our analysis (Fig. 2c). To avoid the influence of filters when estimating the scaling exponent, we thus chose the fitting range sufficiently away from it: between 2 seconds (512 sampling points) and 16 seconds (4096 sampling points; Fig. 2c, Fit Range).

### Stastical Tests

Changes in signal power, DFA scaling exponent and autocorrelation were tested for significance using ANOVA and post hoc t–tests. We also used linear regression based on least-squares fits.

## Results

### Distinct changes in EEG frequency power and power spectrum slope during sleep deprivation

Extended wakefulness is known to be associated with distinct changes in EEG power during waking and consecutive recovery sleep^41–44^. In the wake EEG, the increase in slow frequency power in the theta range (4–8 Hz) is a particularly well-established sleep deprivation hallmark^33^. It has been pointed out that the estimation of long-range temporal correlations (LRTCs) in EEG can, in principle, be affected by EEG power^13,15–17^. We thus started by thoroughly assessing signal power and its changes over the course of sleep deprivation, as this is of central importance for the following characterization of timescales and LRTCs.

We analyzed artifact-free EEG segments of 20-seconds duration during the eyes-open condition. In our data, we observed, in line with previous studies, an increasing signal power in theta as well as in higher frequency ranges including the beta/low gamma range (12–30 Hz; for simplicity referred to as beta in this manuscript) during sleep deprivation (Fig. 1). The significance of power changes over the course of sleep deprivation was evident by linear regression (theta: *r* = 0.80, *p* = 0.0007; beta: *r* = 0.64, *p* = 0.01) and the difference between beginning (0–6 h) and end (33–39 h) of sleep deprivation (theta: *p* = 1*e* − 35; beta: *p* = 4*e* − 10; two-sample t–test). Alpha power (8–12 Hz), conversely, exhibited the well-known circadian modulation^33^ but no significant cumulative power change during sleep deprivation (linear regression: *r* = 0.15, *p* = 0.61; 0–6 hours vs 33–39 hours: *p* = 0.46; Fig. 1b). A heatmap showing the power change in all frequencies across sleep deprivation can be found as Supplementary Fig. S1. Besides these changes in confined frequency bands, we also observed a more shallow slope of the power spectral density (PSD) across a range of lower frequencies after sleep deprivation. The estimation of local slopes from PSD showed that this change to a more shallow slope is significant, and is reversed following recovery sleep (Fig. 1a).

While a large portion of EEG analyses has traditionally focused on restricted frequency bands, the underlying scale-free nature of the EEG PSD has been shown to similarly reveal important information, e.g. with regard to task performance modulation^45,46^. From a dynamical perspective, the scale-free (or 1/f) scaling observed in the PSD may also indicate that the underlying system exhibits long-range spatio-temporal correlations^22^ and slow autocorrelation decay^35^. Interpreted within this framework, changes in the PSD slope, as observed in EEG during sleep deprivation here, could consequently be indicative of changes in the autocorrelation structure and long-range temporal correlations. However, other mechanisms, such as passive filtering, have also been shown to be able to produce scale-free EEG power spectra, even in the absence of any long-range temporal correlations^47^. Methodologically, it can be difficult to obtain robust power spectrum estimates from short time series. For these reasons, the structure of temporal correlations in EEG has been primarily studied in the amplitude envelope of filtered oscillations within confined frequency bands^13,18,19,21,36^.

### Decline in long-range temporal correlations during sleep deprivation

To investigate the existence and the dynamics of long-range temporal correlations during sleep deprivation in our data, we calculated the amplitude envelope of ongoing oscillations in confined frequency bands from all channels of artifact-free EEG segments. We first focused on signal envelopes in the alpha (8–12 Hz) frequency band since average signal power between beginning and end of sleep deprivation did not change significantly there (Fig. 1b). The impact of changes in signal power on LRTC estimates will be studied further below. The autocorrelation function exhibited a faster decay in recordings at the end of sleep deprivation compared to the beginning of sleep deprivation (Fig. 2a). We quantified the decay by the autocorrelation value at time lag-1, which progressively decreased with increasing time awake and recovered towards higher values after consecutive sleep (Fig. 2b). Both changes, the decline during sleep deprivation as well as the increase post recovery sleep, were statistically significant (0–6 hours vs 33–39 hours, *p* = 9*e* − 14; 39 hours vs rec, *p* = 0.0008; two-sample t–test). An alternative quantification of the autocorrelation decay by monitoring the lag when autocorrelation values dropped below 0.5 for the first time exhibited a similar decline during sleep deprivation and can be found in Supplementary Fig. S2.

As a second measure to investigate long-range temporal correlations during the course of sleep deprivation, we used detrended fluctuation analysis (DFA)^48^. A substantial amount of research work has shown that DFA provides a robust measure for the autocorrelation structure of amplitude fluctuations in ongoing neurophysiological oscillations^13,14,16,18,19,21,49^. DFA relies on the quantification of signal fluctuations after removing their trend^36^. It has been suggested that only a comparison of DFA results, using different detrending polynomials, yields full recognition of the trends^20,50^. We thus systematically applied DFA to the same amplitude time series of alpha oscillations as analyzed in the previous section using detrending polynomials from order 1 to 10. Our analysis again revealed a significant decrease of DFA scaling exponents for detrending orders 3 and higher (0–6 hours vs 33–39 hours; two-sample t–test; linear regression analysis; both *p* < 0.05; Fig. 2c,d). Since results did not change qualitatively when detrending was performed with higher order polynomials, we used detrending of 4th order ploynomials (DFA-4) for the remainder of the analyses. We observed DFA scaling exponents in the range of >0.5 to 1, which, in line with previous reports, is indicative of long-range temporal correlations in the EEG signals^13^. Similar to the autocorrelation results we observed a decrease in scaling exponents during sleep deprivation, indicative of a decline in long-range temporal correlations (0–6 hours vs 33–39 hours, *p* = 2*e* − 8; two-sample t–test). The potentially circadian component visible in DFA-4 on top of the decline became less prominent for higher orders of detrending while, at the same time, the DFA scaling exponent for the recovery point became higher, reaching a significantly higher value for detrending order 10 (39 hours vs rec; two-sample t–test; *p* = 0.02).

In principle, the observed changes might be due to both homeostatic (time awake) and circadian factors. For example, the DFA scaling exponent and power in the theta range show both a change with time awake and a superimposed circadian modulation (Figs 1a and 2d). A rested control group to directly disentangle these effects is not feasible with such a sleep deprivation design. However, with 40 h of sustained wakefulness, more than one circadian cycle is covered, thus one can compare measures obtained at the same circadian phase and assess the effect of time awake. We therefore compared autocorrelation measures and DFA exponents over time but within the same circadian phase (i.e. the three recordings from 7:00–13:00 o'clock on day 1 and day 2). The consistently observed decline of all measures is depicted in Supplementary Fig. S3 and thus suggests that the decline is primarily an effect of the time spent awake. Together, these results from the alpha band thus comprehensively indicate a decline in LRTCs with increasing time awake.

### Influence of EEG signal power on long-range temporal correlation estimation

The decline in timescales during sleep deprivation was observed in the alpha band where signal power did not change to overall higher or lower values as a function of time awake. The fact that power did not change is important since timescale estimates from autocorrelation and DFA can, in principle, be influenced by EEG signal power changes, as has been pointed out^13,15–17^. The reasoning behind this argument is that the measured EEG signal always contains some noise component apart from the neuronal signal which, when EEG signal power is lower (higher) can shift estimates of LRTCs to lower (higher) values due to the relative contribution of noise in the signal. A low signal-to-noise ratio can thus lead to lower LRTC values due to the relative contribution of a fast decaying noise autocorrelation function^15^. In line with this conceptual argument, detailed analyses have shown that increasing amounts of noise (or, conversely, decreasing signal-to-noise ratios) can shift autocorrelation and DFA exponents to lower values and vice-versa in EEG^15^. To demonstrate how estimates such as lag-1 autocorrelation can exhibit an apparent dependence on signal amplitude/power when noise is present, we simulated signals composed of a sine wave and a noise term from a uniform distribution. By changing amplitudes of the sine wave and thereby the signal-to-noise ratio, autocorrelation estimates of the signal envelope increased monotonically (Fig. S4a). Conversely, autocorrelation estimates were not effected by different amplitude levels when noise was not present (Fig. S4b). Previous work aware of this issue has thus carefully investigated potential correlations between LRTCs and signal power which were found to be marginal but significant (alpha and beta bands^15,17^) to strong (theta^17^). Under conditions with drastic power changes in relevant frequency bands, such as during sleep deprivation in our data, it is therefore possible that these effects will impact the estimation of LRTCs.

**Figure 3:**
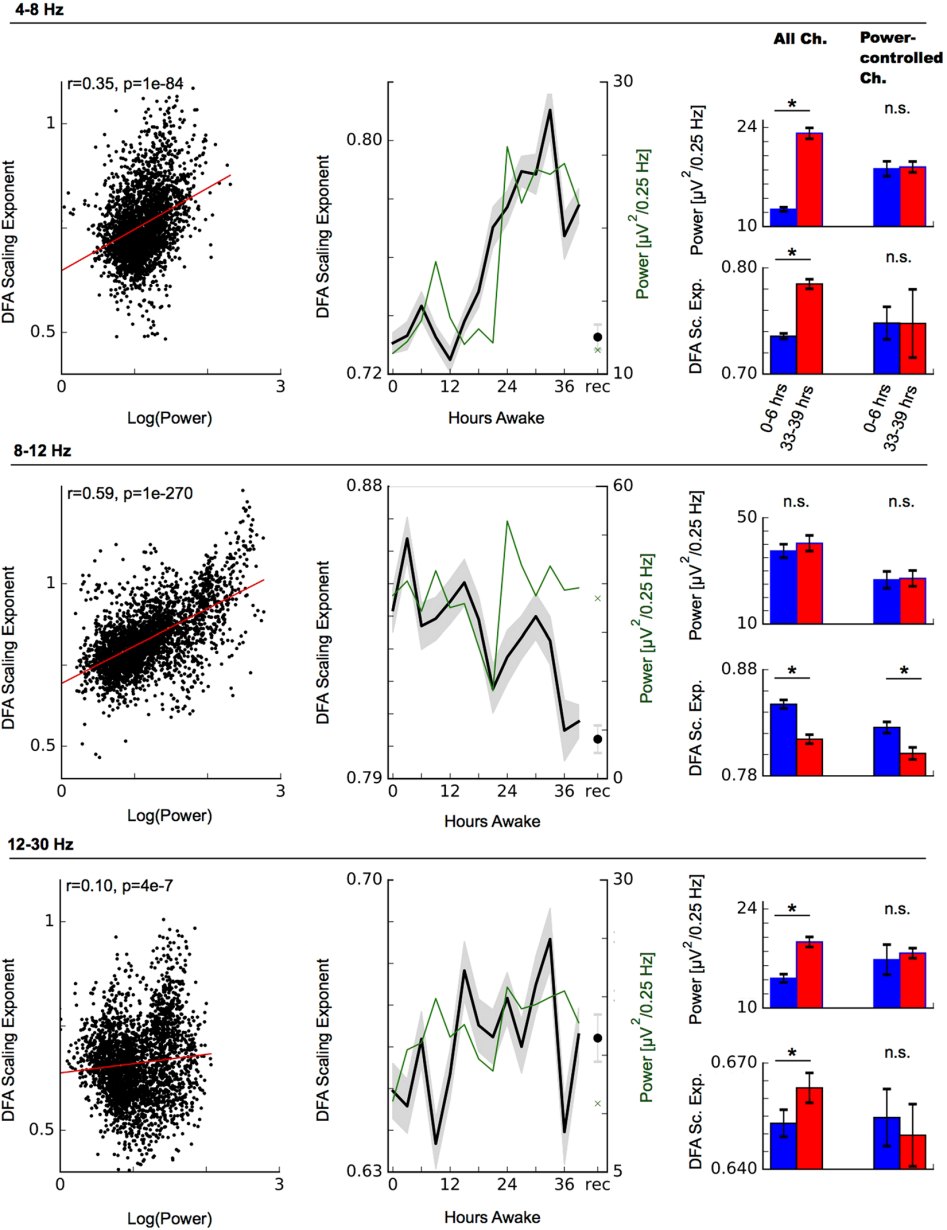
Influence of signal power on estimating long-range temporal correlations. DFA scaling exponents exhibited a significant correlation with signal power in all frequency bands investigated (left column). Middle column: DFA scaling exponents (black) follow a very similar trajectory to signal power (green) particularly when power is changing significantly, as is the case in the theta (top) and beta (bottom) bands. Right column: DFA increases between early (0–6 hours, blue) and late (33–39 hours, red) sleep deprivation vanish in the theta and beta bands when only channels with no significant power change (power controlled channels) are considered for DFA estimation. Conversely, the decline in DFA scaling exponents in the alpha band remains. This suggests that it is the strongly increasing signal power in theta and beta bands that leads to an apparent increase of DFA scaling exponents in these frequency bands.

In our data we observed that DFA scaling exponents were significantly correlated with signal power in all three frequency bands (Fig. 3 left column). In stark contrast to the alpha band, we observed an increase in the DFA scaling exponent in the theta band which closely mirrored the time course of theta power (Fig. 3 top). Specifically, the DFA exponent exhibited a circadian component on top of a monotonic increase which closely resembles the well-known increase of theta power during sleep deprivation^33^. This striking similarity in time course along with the significant correlation between power and scaling exponent thus suggest that the increase in DFA exponents in theta may be primarily due to the concomittant changes in signal power levels. This influence of signal power is also likely to determine the time course of DFA scaling exponents in the beta band to a large extent where signal power increases significantly too and may similarly impact the signal-to-noise ratio and estimation of LRTCs^15^.

For better insight into the influence of the strongly increasing power on DFA exponents in theta and beta frequency bands, we repeated the analysis using only channels that did not show a significant change (0–6 hour vs 33–39 hour; two-sided t–test; *p* > 0.5). Results were similar when different p-values (0.3–0.9) were used to exclude channels with significant power changes. By controlling for power changes (and in particular power increases) during sleep deprivation with this method, we observed no significant increases in DFA scaling exponents in both theta (7/189 remaining channels) and beta bands (31/189 channels remaining; 0–6 hours vs 33–39 hours; *p* > 0.05; two-sample t–test; Fig. 3 right column). In fact, in theta band, scaling exponents even exhibited a slight decrease, albeit not reaching the significance level. When we controlled for power changes in the same way in the alpha band (87/189 channels remaining), the significant decrease in DFA scaling exponents during sleep deprivation persisted (*p* = 0.0008; two-sample t–test). Together, these analyses thus provide strong indication that the apparent increase of LRTCs in theta and beta bands is due to the concomittant increase in signal power. Conversely, when no significant power changes are present, such as in the alpha band, then LRTCs decline during sleep deprivation.

### LRTC decline is predominantly found in frontal and parieto-occipital regions

**Figure 4:**
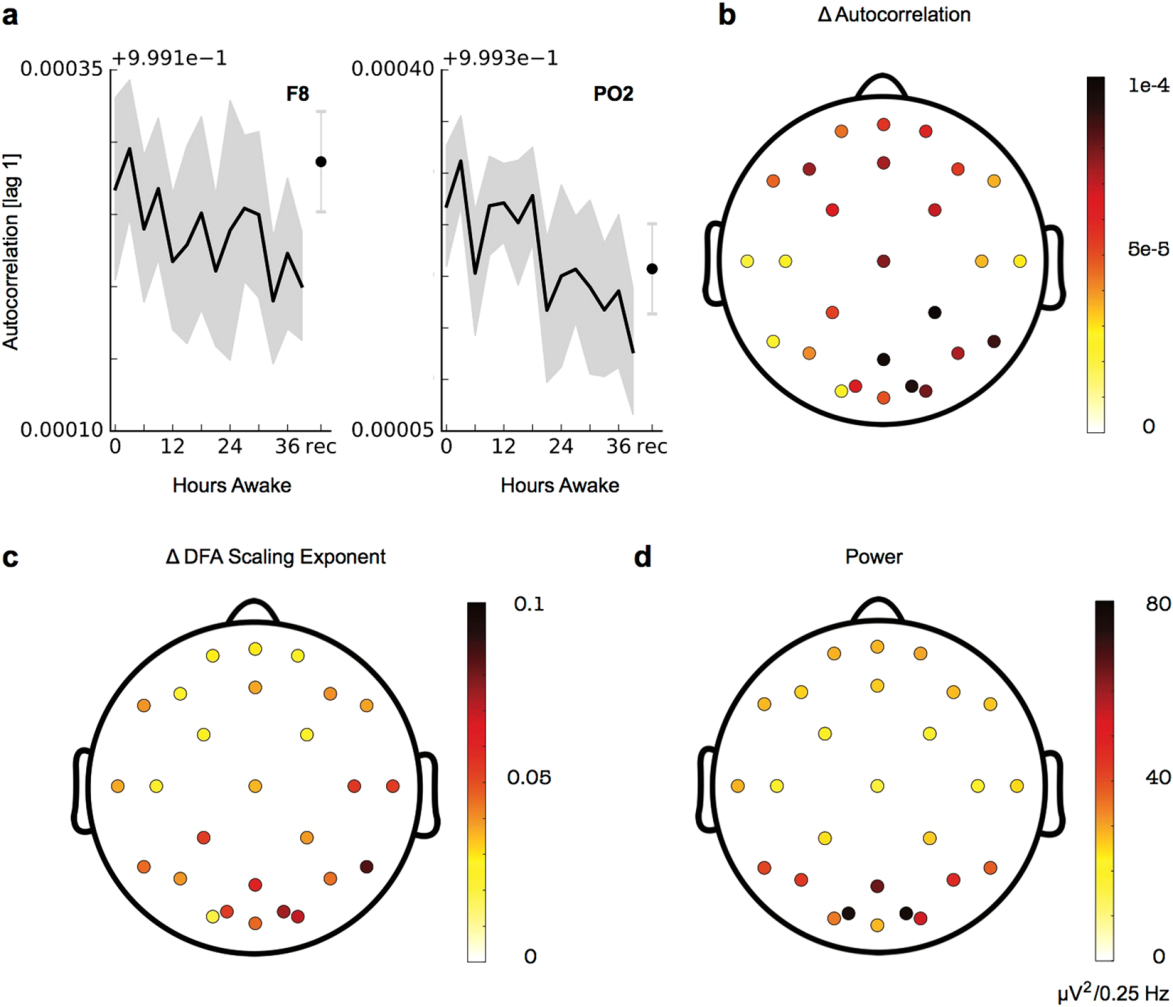
The decline of timescales during sleep deprivation occurs over broad cortex areas with a particular dominance in frontal and parieto-occipital regions. All plots correspond to results from the alpha band (8–12 Hz). (**a**) Exemplary time courses from two individual EEG channels indicating a strong decline in autocorrelation values during sleep deprivation. (**b**) Topographic distribution of changes in lag-1 autocorrelation during sleep deprivation (difference between average values from 0–6 hours and average values from 33–39 hours). Each marker corresponds to one EEG channel. While a decrease in autocorrelation is evident in all channels, changes a particularly prominent in frontal and parieto-occipital regions. (**c**) Topographic distribution of sleep deprivation changes in DFA scaling exponents. (**d**) Topographic distribution of average power in the alpha band.

Our described trends so far were based on averages across all channels. The decline in LRTC could also be observed at the individual channel level (Fig. 4a; alpha band). The topographical distribution of sleep deprivation changes (0–6 hours vs 33–39 hours) showed that LRTC decreases are visible across all EEG channels and are particularly prominent over frontal and parieto-occipital leads for both autocorrelation and DFA (Fig. 4b,c).

## Discussion

In the present work, we report the decline of long-range temporal correlations (LRTCs) in cortical activity during sustained wakefulness. We estimated the timescales of temporal correlations in EEG amplitude fluctuations of ongoing neuronal oscillations from the autocorrelation function directly, as well as using detrended fluctuation analysis. While both analyses indicated the presence of long timescales associated with a slow autocorrelation decay at the beginning of sleep deprivation, sustained wakefulness led to a progressive decline with shorter timescales. We observed this decline of LRTCs to be visible when measures were obtained from the same circadian phase which suggests the leading cause to be the time spent awake. Given that EEG recordings were relatively short, other causes, such as for example time-on-task or boredom, are rather unlikely. Our results provide a novel perspective on the changes of cortical network dynamics with implications for their information processing capabilities during sustained wakefulness. They provide a missing link to previous findings indicating a disruption of critical dynamics during sleep deprivation^32^ and highlight the importance of adequately taking into consideration signal power changes when assessing long-range temporal correlations in EEG.

Our main analyses focused on LRTCs in the alpha band where signal power exhibits a circadian modulation, but no significant overall in- or decrease occurs as a function of time awake. Under these conditions, a clear decline in LRTCs quantified by autocorrelation directly and by detrended fluctuation analysis could be observed during sustained wakefulness. Conversely, in the other frequency bands studied (theta and beta), signal power increased significantly over the course of sleep deprivation which compromised the estimation of LRTCs in these bands. The relevance of signal power for the estimation of LRTCs has been pointed out and studied several times before^15–17^. Broadly speaking, estimates of LRTCs can be biased towards lower, more noise-like values when signal power (and thus the signal-to-noise ratio) is lower and vice-versa. Consequently, Linkenkaer-Hansen *et al*. observed positive, albeit weak, correlations between signal power and DFA exponents in some EEG channels^15^. In a similar vein, Smit *et al*. reported marginal correlations of LRTCs with power in the alpha and beta bands and substantial correlations in the theta band, which led the authors to conclude that the stronger signal-to-noise ratio in this frequency band may have caused increased LRTCs^17^. Our results confirm this dependence and indicate that these effects are particularly important to consider in experimental conditions when signal power changes dramatically in some frequency bands, such as during sleep deprivation^33^. To make a robust conclusion about changes in LRTCs, or any other EEG measure for that matter, in an experiment, it is therefore important to carefully consider whether these changes could be caused by concomitant alterations in signal power. Here, we observed decreasing LRTCs during sleep deprivation when power remained constant (alpha band) and an apparent increase in the other bands (beta and theta) only when there was a drastic concomitant power increase. This is similar to our observations of synchronization measures in a previous study^32^, which were also found to be most predominant in the alpha band. Thus, these changes in LRTCs and synchronization measures cannot be explained as a direct consequence of the alterations in spectral power, which are most evident in the theta band^33^.

Differences in signal power and signal-to-noise ratio may potentially also help to reconcile some perhaps contradictory findings. In a recent study, larger LRTCs associated with higher insomnia complaints were observed within groups of insomnia patients and control subjects and interpreted as a sign of being closer to criticality^51^. Interestingly, no LRTC differences were observed between control and insomnia patient groups. In light of the strong impact of signal power on estimates of LRTCs in our data, especially under conditions with high sleep pressure, it is possible that signal power changes associated with worse sleep quality may have contributed to the apparent increase in LRTCs in these subjects, similar to our data.

The extended timescales and memory effects associated with LRTCs have long been thought to provide favorable neuronal substrates for the integration of information across time and across different cortical areas in order to increase the signal-to-noise ratio in cognitive tasks, such as, for example, during decision making^7,9,11,13^. This notion is supported by several experimental studies where LRTCs were found to correlate with and predict behavioral performance^19,21^. In the present work we link changes in these dynamical signatures to sleep deprivation for which cognitive impairments are well known^1–4^, but the corresponding neuronal correlates have thus far been difficult to identify. Our work suggests the long cortical timescales and LRTCs as promising candidates for these neuronal correlates. The question whether cortical timescales can also predict cognitive function over the course of sleep deprivation will be the topic of future research. Future studies may help to substantiate the generalizability of our findings with respect to larger subject cohorts and cohorts consisting of both women and men and thereby overcome the limitations of the present study in terms of the small sample size (8 subjects) and the absence of cognitive correlates. A very recent report of similar cortical timescale declines in individual neuron activity in rodents during sleep deprivation suggests this framework to be applicable across scales and species to describe network dynamics in terms of its ability to integrate information over time^52^.

Long-range temporal correlations are generic features of systems in the vicinity of a critical state^22^. The observation of LRTCs in EEG has thus been taken as additional evidence for a growing body of computational and experimental studies indicating that cortical neural networks operate at some sort of critical state^13,24,26–28,30,31^. Interpreted within this criticality framework, the decline of LRTCs during sustained wakefulness complements the observed fading of other signatures of critical dynamics during extended wake^32^. This framework also provides an interesting link between network function and the often-observed impairments of cognitive capabilities during extended wake. Critical dynamics are often regarded to support optimal computational functioning^23–26,53^. The decline of LRTCs and other signatures of critical dynamics during prolonged wakefulness suggests that the brain can benefit less from the computational advantages of critical or near-critical dynamics. Behavioral observations of impaired cognitive functioning and information processing after sleep deprivation^2^ might be the result of these functional deficits.

With regard to the mechanisms responsible for the observed disruption of long timescales, it is conceivable that the control of cortex dynamics through subcortical regions might play a role. It is known that several subcortical structures in the brainstem, hypothalamus and basal forebrain regulate the maintenance of waking and sleep states through neuro-modulatory action^54^. During extended wake, these subcortical structures could transiently disrupt the ongoing cortical dynamics leading to an apparent decline in long-range temporal correlations. Alternatively, it is conceivable that sleep/wake dependent structural changes in cortical networks, such as changes in average synaptic strenghts^55,56^, might drive network dynamics closer/further away from criticality and thereby effect cortical timescales. Independent of the underlying mechanisms, our results indicate that sleep is important to reorganize cortical networks towards dynamics with long-range temporal correlations for optimal function during wake.

## Electronic supplementary material

Supplementary Information
