# Supplementary material for: Decline of long-range temporal correlations in the human brain during sustained wakefulness
Source: Sci Rep. 2017 Sep 19;7:11825. doi: 10.1038/s41598-017-12140-w (PMC5605531; doi:10.1038/s41598-017-12140-w)
Supplement: Supplementary file 1 — Supplementary Information [file 41598_2017_12140_MOESM1_ESM.pdf]

Supplementary Information

Decline of long-range temporal correlations in the human brain during sustained wakefulness

Christian Meisel[1,2], Kimberlyn Bailey[1], Peter Achermann[3] and Dietmar Plenz[1]

1 Section on Critical Brain Dynamics, National Institute of Mental Health, Bethesda, Maryland 20892, USA
2 Department of Neurology, University Clinic Carl Gustav Carus, Fetscherstrasse 74, 01307 Dresden, Germany
3 Institute of Pharmacology and Toxicology, University of Zurich, Winterthurerstrasse 190, 8057 Zurich, Switzerland

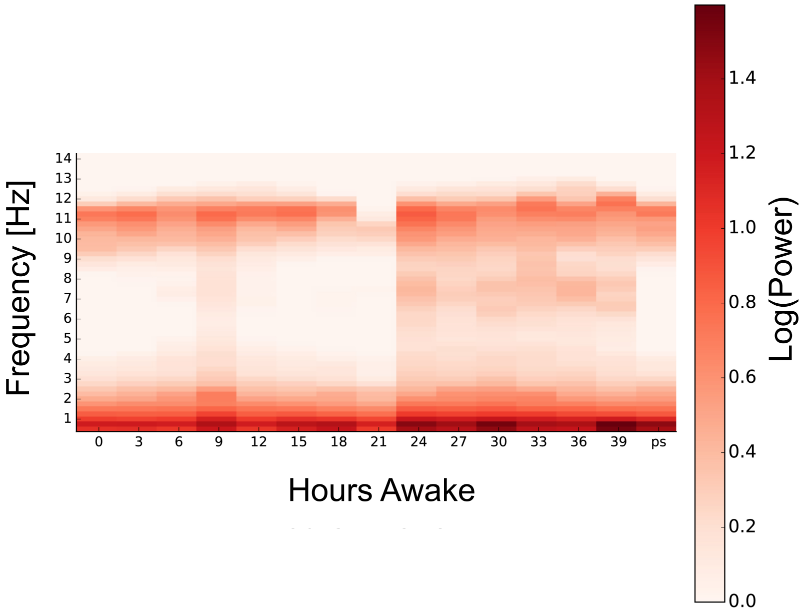

Figure S1. Heatmap of EEG signal power across sleep deprivation and post recovery sleep (ps). Values refer to averages across all EEG channels and all 8 subjects.

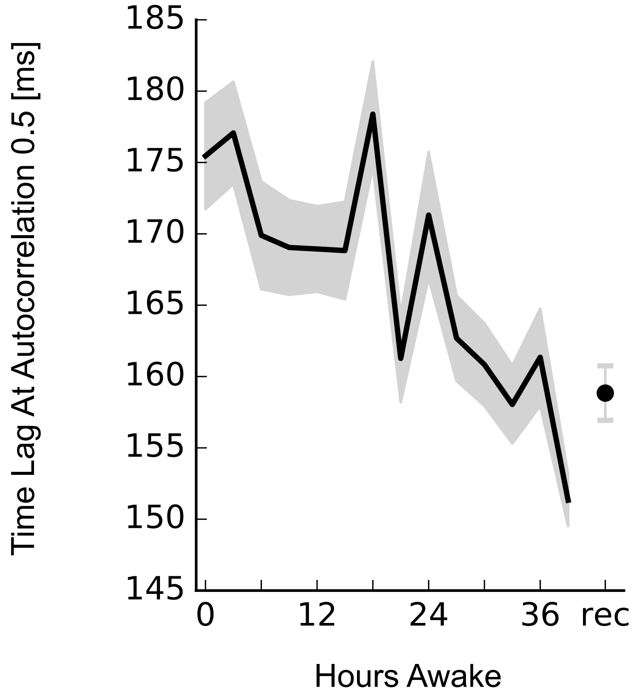

Figure S2. Decline in signal envelope autocorrelation in the alpha band (8-12 Hz) during sleep deprivation quantified in an alternative way. As an alternative method to capture the faster decay of the autocorrelation function in comparison to the results presented in the main part of the manuscript, the time lag at which the autocorrelation function crosses below the value of 0.5 from above for the first time is depicted here. Consequently, a shorter time lag is observed as the autocorrelation function decays faster. The solid black line corresponds to the mean across all channels from all 8 subjects, error bars indicate s.e.m. The decline of time lags during sleep deprivation is statistically significant (0-6 hours vs 33-39 hours; ANOVA and post hoc two-sample t-test; $p<4e-11$).

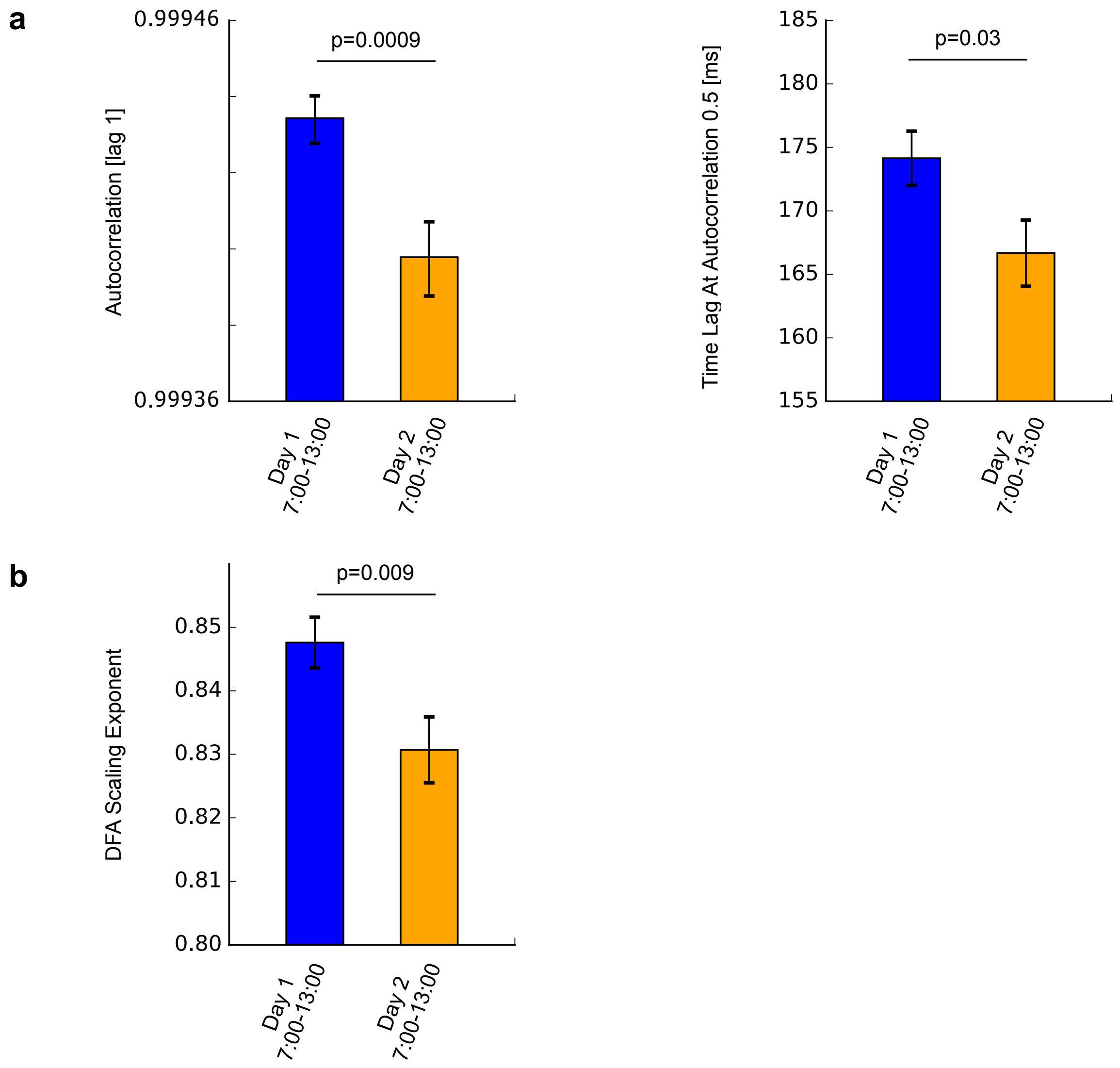

Figure S3. Declines of autocorrelation and DFA scaling exponents are observed when measures are compared within the same circadian phase. In principle, the overall observed changes in LRTCs might be due to both homeostatic (time awake) and circadian factors. However, as with 40 h of sustained wakefulness more than one circadian cycle is covered, one can compare measures obtained at the same circadian phase (i.e. the three recording from 7:00-13:00 o'clock on day 1 and day 2) and thus assess the effect of time awake. a, Decline of autocorrelation measured in two alternative ways. b, Decline of DFA scaling exponent over the course of sleep deprivation. P-values refer to two-sample t-tests.

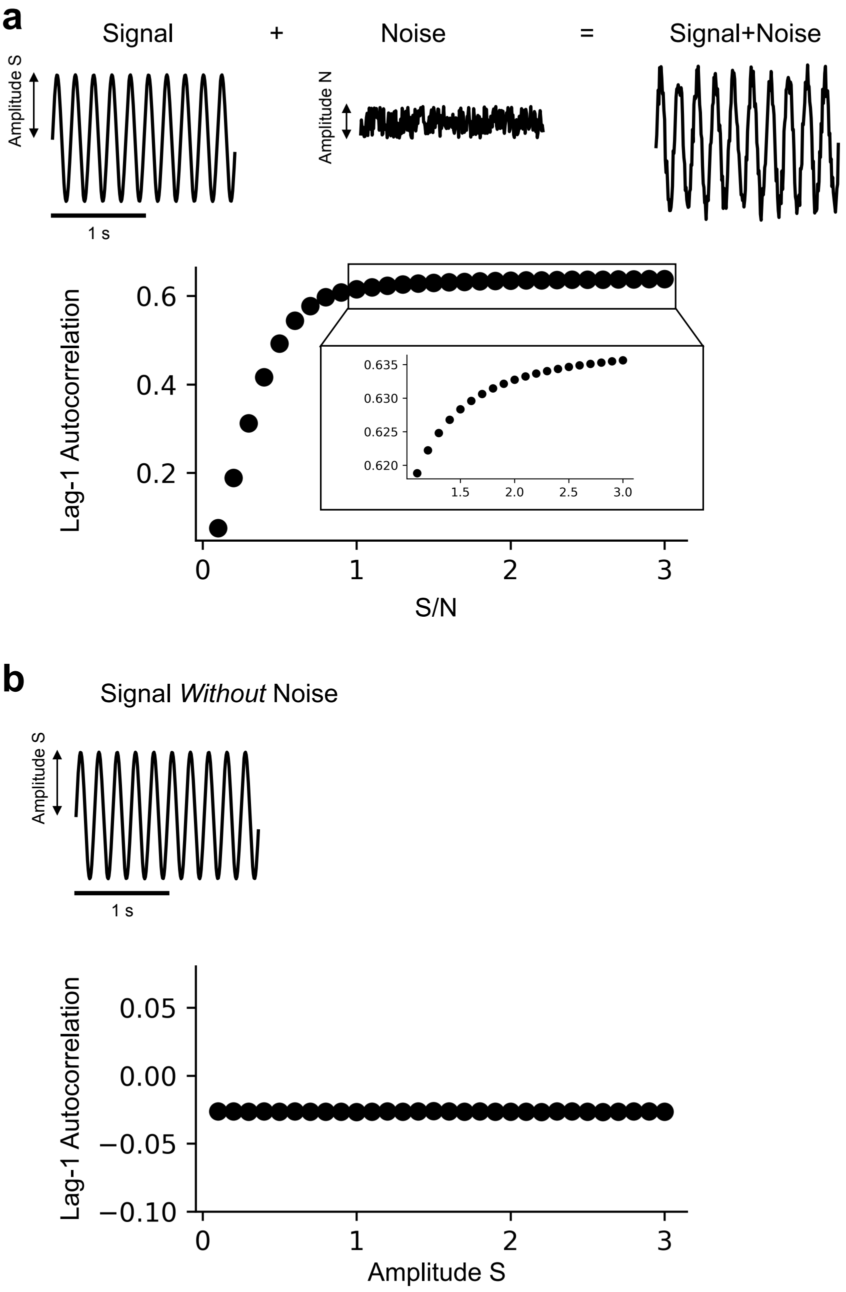

Figure S4. Schematic demonstration how the ratio of signal to noise can impact estimates of long-range temporal correlations, such as lag-1 autocorrelation.
a, Top: for demonstration purposes, consider a simulated signal composed of a sine wave with amplitude S and a uniform noise term with amplitude N. The data under investigation for potential long-range temporal correlations (lag-1 autocorrelation of the data's Hilbert transform in this case) is thus composed of these two terms, signal and noise. Bottom: the plot shows the lag-1 autocorrelation of this data composed of signal and noise for different ratios of signal S and noise N amplitudes (inset: zoomed in plot for the S/N range from 1 to 3). There exists is a positive relationship between autocorrelation values and S/N. A lower/higher signal amplitude S relative to a constant remaining noise amplitude N can therefore lead to lower/higher autocorrelation estimates. For a similar analysis for DFA exponents, see Fig. 3 in Linkenkaer et al., 2007.
b, When no noise is present, lag-1 autocorrelation estimates are independent of signal amplitude.

Reference:
Linkenkaer-Hansen, K. *et al.* Genetic contributions to long-range temporal correlations in ongoing oscillations. *J. Neurosci.* 27, 13882–13889 (2007).
